# Neighborhood Deprivation and Incident Alzheimer's Disease: A Regional Cohort Study of Electronic Medical Records

**DOI:** 10.1093/geroni/igab046.230

**Published:** 2021-12-17

**Authors:** Jarrod Dalton, Elizabeth Pfoh, Kristen Berg, Douglas Gunzler, Lyla Mourany, Nikolas Krieger, Eva Kahana, Adam Perzynski

**Affiliations:** 1 Cleveland Clinic and Case Western Reserve University, Cleveland, Ohio, United States; 2 Cleveland Clinic, Cleveland, Ohio, United States; 3 Case Western Reserve University at MetroHealth, Cleveland, Ohio, United States; 4 Case Western Reserve University, Cleveland, Ohio, United States

## Abstract

The prevalence of Alzheimer’s disease (AD) is anticipated to increase drastically. Neighborhood socioeconomic position (SEP) has been related to multiple processes of health. Understanding whether SEP is related to AD can inform who is at greatest risk of developing this disease. We analyzed electronic medical records of 394892 patients from the two largest health systems in Northeast Ohio to evaluate the relationship between Ohio Area Deprivation Index quintiles (defined at the census tract level) and hazard for a composite outcome of AD diagnosis or primary AD death. We included residents of Cuyahoga and neighboring counties, and used the first outpatient visit beyond age 60 occurring between 2005 and 2015 as study baseline. Outcome data were censored at the earlier of a) the beginning of any 3-year time period without visits or b) non-AD death. We estimated a Cox proportional hazards regression model, adjusting ADI quintile effects for the interaction between age at baseline, sex and race as well as birth year. We used quadratic terms for continuous predictors. After adjusting for these factors, ADI quintile was significantly related (χ2 = 83.0 on 4 d.f.; p < 0.0001) to the composite time-to-event outcome. Compared to the lowest-deprivation quintile, ADI quintiles 4 (adjusted hazard ratio [95% confidence interval]: 1.18 [1.10, 1.26]) and 5 (1.37 [1.28, 1.47]) had significantly higher hazard for the composite outcome. In conclusion, neighborhood deprivation may be a risk factor for AD independent of demographic factors. Preventive efforts should target individuals living in neighborhoods with high levels of deprivation.

